# Lemongrass (*Cymbopogon flexuosus*) essential oil demonstrated anti-inflammatory effect in pre-inflamed human dermal fibroblasts

**DOI:** 10.1016/j.biopen.2017.03.004

**Published:** 2017-03-21

**Authors:** Xuesheng Han, Tory L. Parker

**Affiliations:** dōTERRA International, LLC, 389 S. 1300 W., Pleasant Grove, UT 84062, USA

**Keywords:** Lemongrass essential oil, Inflammation, Tissue remodeling, Genome-wide gene expression, Vascular cell adhesion molecule 1, Interferon gamma-induced protein 10

## Abstract

Lemongrass (*Cymbopogon flexuosus*) essential oil (LEO), which has citral as its main component, has exhibited anti-inflammatory effect in both animal and human cells. In this study, we evaluated the anti-inflammatory activity of a commercially available LEO in pre-inflamed human dermal fibroblasts. We first studied the impact of LEO on 17 protein biomarkers that are critically associated with inflammation and tissue remodeling. LEO significantly inhibited production of the inflammatory biomarkers vascular cell adhesion molecule 1 (VCAM-1), interferon gamma-induced protein 10 (IP-10), interferon-inducible T-cell alpha chemoattractant (I-TAC), and monokine induced by gamma interferon (MIG); decreased levels of the tissue remodeling biomarkers collagen-I and III, epidermal growth factor receptor (EGFR), and plasminogen activator inhibitor (PAI-1); and inhibited the immunomodulatory biomarker macrophage colony-stimulating factor (M-CSF). Furthermore, we studied the impact of LEO on genome-wide gene expression profiles. LEO significantly modulated global gene expression and robustly impacted signaling pathways, many of which are critical for inflammation and tissue remodeling processes. This study provides the first evidence of the anti-inflammatory activity of LEO in human skin cells and indicates that it is a good therapeutic candidate for treating inflammatory conditions of the skin.

## Highlights

• Lemongrass essential oil (LEO) was antiproliferative to human dermal fibroblasts.
• LEO inhibited the inflammatory molecules VCAM-1, IP-10, I-TAC, and MIG.
• LEO inhibited the tissue-remodeling molecules collagen-I and III, EGFR, and PAI-1.
• LEO inhibited the immunomodulatory molecule M-CSF.
• LEO impacted genome-wide gene expression profiles in human skin cells.

## 1 Introduction

Lemongrass (*Cymbopogon flexuosus*) essential oil (LEO^1^) has been traditionally used as a remedy for a variety of health conditions. Recent scientific studies have provided evidence supporting its antimicrobial, antioxidant, antifungal, and anti-inflammatory properties in several disease models [1], [2], [3], [4], [5], [6]. Boukhatem et al. showed that topical application of LEO inhibits the skin inflammatory response in mice [2]. Jiang et al. found that LEO protected against benzo-α-pyrene-induced oxidative stress and DNA damage in human embryonic lung fibroblast cells [6]. A recent cytotoxicity study in human dermal fibroblasts by Adukwu et al. determined the IC_50_ to be 0.126% (*v*/*v*) for LEO and 0.095% (*v*/*v*) for citral, the primary component of LEO [5].

Given the popularity of topically applied LEO and the lack of bioactivity study in human skin cells, we undertook an investigation of the biological activity of a commercially available LEO in a validated human skin cell culture system. We first studied its impact on 17 protein biomarkers that are closely related to inflammation and tissue remodeling processes. We also studied its effect on modulating human genome-wide gene expression profiles. The results showed that LEO significantly inhibited the production of many inflammatory biomarkers and robustly impacted global gene expression profiles in human skin cells.

## 2 Materials and methods

All experiments were conducted using a BioMAP HDF3CGF system, which was designed to model the pathology of chronic inflammation in a robust and reproducible manner. The system comprises three components: a cell type, stimuli to create the disease environment, and a set of biomarker (protein) readouts to examine how the treatments affected the disease environment [7]. The methodologies used in this study were essentially the same as those previously described [8], [9], [10].

### 2.1 Cell culture

Primary human neonatal fibroblasts were prepared as previously described [11] and were plated under low serum conditions for 24 h before stimulation with a mixture of interleukin (IL)-1β, tumor necrosis factor (TNF)-α, interferon (IFN)-ϒ, basic fibroblast growth factor (bFGF), epidermal growth factor (EGF), and platelet-derived growth factor (PDGF). The cell culture and stimulation conditions for the HDF3CGF assays have been described in detail elsewhere [11].

### 2.2 Protein-based readouts

Enzyme-linked immunosorbent assay (ELISA) was used to measure the biomarker levels of cell-associated and cell membrane targets. Soluble factors in the supernatants were quantified using homogeneous time-resolved fluorescence detection, bead-based multiplex immunoassay, or capture ELISA. The adverse effects of the test agents on cell proliferation and viability (cytotoxicity) were measured using the sulforhodamine B (SRB) assay. For proliferation assays, the cells were cultured for 72 h before measurements, which is optimal for the HDF3CGF system. The detailed procedure has been described in a previous study [11]. Measurements were performed in triplicate, and a glossary of the biomarkers used in this study is provided in Supplementary Table S1.

Quantitative biomarker data are presented as the mean log_10_ relative expression level (compared to the respective mean vehicle control value) ± standard deviation (SD) of triplicate measurements. Differences in biomarker levels between LEO- and vehicle-treated cultures were tested for significance using the unpaired Student's t test. A p-value < 0.05, outside of the significance envelope, with an effect size of at least 10% (>0.05 log_10_ ratio units), was regarded as statistically significant.

### 2.3 RNA isolation

Total RNA was isolated from cell lysates using the Zymo *Quick-RNA* MiniPrep kit (Zymo Research Corp., Irvine, CA, USA) according to the manufacturer's instructions. RNA concentration was determined using a NanoDrop ND-2000 system (Thermo Fisher Scientific). RNA quality was assessed using a Bioanalyzer 2100 (Agilent Technologies, Santa Clara, CA, USA) and an Agilent RNA 6000 Nano kit. All samples had an A260/A280 ratio between 1.9 and 2.1 and a RNA Integrity Number score >8.0.

### 2.4 Microarray analysis for genome-wide gene expression

A 0.0012% (v/v) concentration of LEO was tested for its effect on the expression of 21,224 genes in the HDF3CGF system after a 24 h treatment. Samples for microarray analysis were processed by Asuragen, Inc. (Austin, TX, USA) according to the company's standard operating procedures. Biotin-labeled cRNA was prepared from 200 ng of total RNA using an Illumina TotalPrep RNA Amplification kit (Thermo Fisher Scientific) and one round of amplification. The cRNA yields were quantified using ultraviolet spectrophotometry, and the distribution of the transcript sizes was assessed using the Agilent Bioanalyzer 2100. Labeled cRNA (750 ng) was used to probe Illumina human HT-12 v4 expression bead chips (Illumina, Inc., San Diego, CA, USA). Hybridization, washing, staining with streptavidin-conjugated cyanine-3, and scanning of the Illumina arrays were carried out according to the manufacturer's instructions. The Illumina BeadScan software was used to produce data files for each array; raw data were extracted using Illumina BeadStudio software.

The raw data were uploaded into R [12] and analyzed for quality-control metrics using the beadarray package [13]. The data were normalized using quantile normalization [14], and then re-annotated and filtered to remove probes that were non-specific or mapped to intronic or intragenic regions [15]. The remaining probe sets comprised the dataset for the remainder of the analysis. The fold-change expression for each set was calculated as the log_2_ ratio of LEO to the vehicle control. These fold-change values were uploaded onto the Ingenuity Pathway Analysis web software (IPA, QIAGEN, Redwood City, CA, USA, www.qiagen.com/ingenuity) to generate the networks and pathway analyses.

### 2.5 Reagents

LEO (dōTERRA International LLC, Pleasant Grove, UT, USA) was diluted in DMSO to 8× of the final concentrations (final DMSO concentration in culture media was no more than 0.1% [v/v]); 25 μL of each 8× solution was added to the cell culture to a final volume of 200 μL. DMSO (0.1% [v/v]) served as the vehicle control. The gas chromatography–mass spectrometry analysis of LEO indicated that its major chemical constituents (i.e., >5%) were geranial (also known as citral A) (43%), neral (also known as citral B) (32%), and geraniol (6%).

## 3 Results and discussion

### 3.1 Bioactivity profile of LEO in the HDF3CGF system

**Fig. 1:**
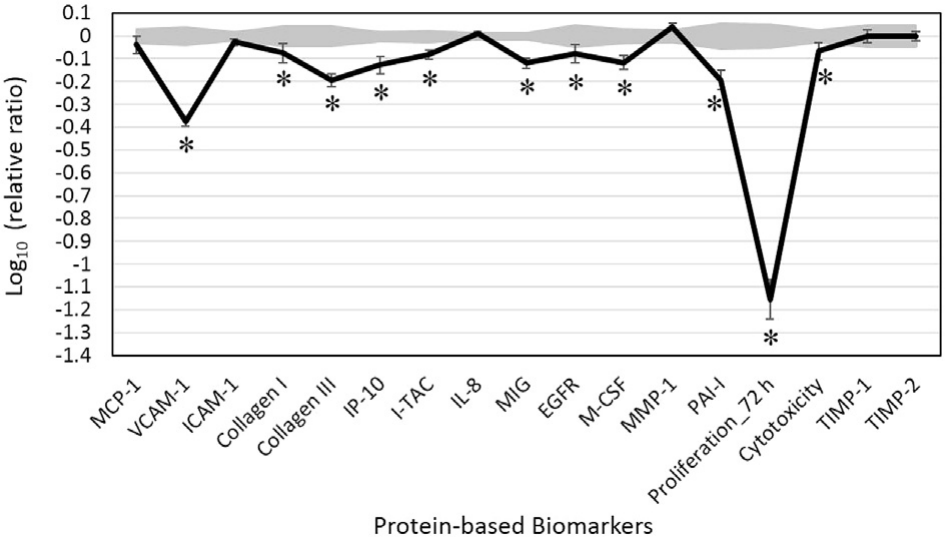
Bioactivity profile of lemongrass essential oil (LEO, 0.0012% v/v) on the human dermal fibroblast culture system HDF3CGF. X-axis denotes protein-based biomarker readouts. Y-axis denotes relative expression levels of biomarkers compared to vehicle control values. Vehicle control values are shaded gray, with 95% confidence levels. A * indicates key biomarkers, whose expression was significantly different (p < 0.05) from vehicle controls at the studied concentration, with an effect size of at least 10% (more than 0.05 log ratio units). MCP-1, monocyte chemoattractant protein; VCAM-1, vascular cell adhesion molecule 1; ICAM-1, intracellular cell adhesion molecule 1; IP-10, interferon gamma-induced protein 10; I-TAC, interferon-inducible T-cell alpha chemoattractant; IL-8, interleukin-8; MIG, monokine induced by gamma interferon; EGFR, epidermal growth factor receptor; M-CSF, macrophage colony-stimulating factor; MMP-1, matrix metalloproteinase 1; PAI-1, plasminogen activator inhibitor 1; TIMP, tissue inhibitor of metalloproteinase.

We analyzed LEO's activity in the validated dermal fibroblast system, HDF3CGF, which features the microenvironment of inflamed human skin cells. Four different concentrations (0.011%, 0.0037%, 0.0012%, and 0.00041%, v/v) of LEO were initially tested for cytotoxicity. LEO was cytotoxic to the cells at concentrations of 0.011% and 0.0037%. Therefore, a concentration of 0.0012% was included for further analysis. Biomarkers with significantly different expression (p < 0.05) compared to that of vehicle controls, with an effect size of at least 10% (>0.05 log ratio units) (Fig. 1), were considered important. The details are discussed below.

LEO showed significant inhibition of 11 of the 17 studied protein biomarkers. LEO exhibited significant anti-proliferative activity in dermal fibroblast cells. LEO significantly decreased production of several inflammatory biomarkers, including vascular cell adhesion molecule 1 (VCAM-1), interferon gamma-induced protein 10 (IP-10), interferon-inducible T-cell alpha chemoattractant (I-TAC), and monokine induced by gamma interferon (MIG). Tissue remodeling biomarkers collagen-I and III, epidermal growth factor receptor (EGFR), and plasminogen activator inhibitor (PAI-1) were also significantly inhibited by LEO. LEO also significantly inhibited the production of macrophage colony-stimulating factor (M-CSF), an immunomodulatory biomarker.

LEO typically has very high amount of citral (geranial and neral). The anti-inflammatory property of LEO has been largely attributed to the activity of citral. Boukhatem et al. found that both topical and oral administration of LEO significantly inhibited chemically induced skin inflammation in a mouse model [2]. Another group also showed that LEO elicited significant anti-allergic and anti-inflammatory effects in a mouse edema model [16]. Both of these reports investigated LEO with a chemical composition similar to that of the LEO used in the present study. Amorim et al. evaluated the anti-inflammatory property of four citrus essential oils, and found that *Cymbopogon aurantifolia* essential oil, largely due to high levels of citral, significantly reduced carrageenan-induced inflammation in the subcutaneous air pouch model [3]. An animal study of citral showed that it significantly inhibited oxidative stress, apoptosis, and macrophage and nuclear factor-kB activation, demonstrating beneficial action through antioxidant and anti-inflammatory activities [17]. Song et al. recently found that citral significantly inhibited enhanced production of TNF-α, IL-8, VCAM-1, and ICAM-1 in human umbilical vein endothelial cells [18]. Consistent with these existing studies, the current finding of the inhibitory effect of LEO on inflammatory biomarkers indicates that it may possess anti-inflammatory properties and modulate the tissue remodeling process in pre-inflamed human dermal fibroblast cells. Moreover, the anti-inflammatory activity of LEO, along with its anti-proliferative effect in human skin cells, might promote enhanced wound healing, presumably via acceleration of proper tissue remodeling processes [19].

### 3.2 Effects of LEO on genome-wide gene expression profiles in the HDF3CGF system

To further explore the biological activities of LEO, we studied the effect of 0.0012% LEO (the highest non-cytotoxic concentration) on the RNA expression of 21,224 genes in the HDF3CGF system. The results showed robust and diverse effects of LEO on human gene regulation. Among the 200 most impacted genes (log_2_ [expression fold-change ratio relative to vehicle control] ≥ |1.5|), the majority (135 out of 200 genes) were significantly inhibited (Table S2). A cross-comparison of protein and gene expression data revealed that VCAM-1 was among those most inhibited by LEO at both protein and gene levels. See Supplementary Material for more information.

**Fig. 2:**
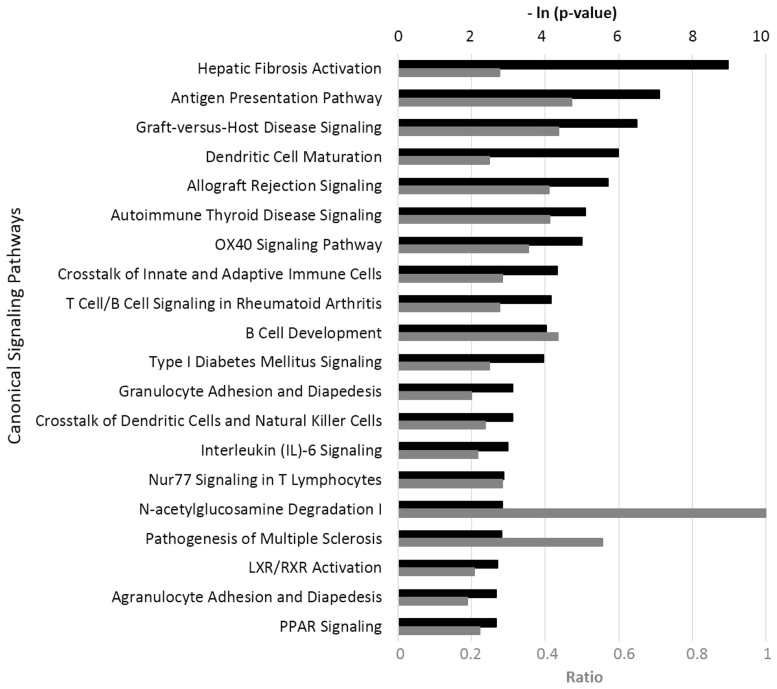
Top 20 canonical pathways representing the effect of lemongrass essential oil (LEO, 0.0012% v/v) on gene expression in the HDF3CGF system, generated via IPA. The p-value is calculated with the right-tailed Fisher's Exact Test. The p-value measures how likely the observed association between a specific pathway and the dataset would be if it was only due to random chance. The smaller the p-value (the bigger the −ln (p-value), indicated by black bars) of a pathway, the more significantly it matches with the bioactivity of LEO. A ratio, indicated by the gray bar, is calculated by taking the number of genes from the LEO dataset that participate in a canonical pathway, and dividing it by the total number of genes in that pathway. OX40, tumor necrosis factor receptor superfamily, member 4; Nur77, nuclear hormone receptor 77; LXR, liver X receptor; RXR, retinoid X receptor; PPAR, peroxisome proliferator-activated receptor.

Further IPA studies showed that the bioactivity of LEO significantly overlapped with many canonical signaling pathways from the literature-validated database (Fig. 2). It is noteworthy that many of the most impacted pathways are closely related to processes of inflammation and tissue remodeling in human cells (Fig. 2, Tables S3–S6). LEO showed an overall inhibitory effect on these critical genes and signaling pathways, consistent with its anti-inflammatory activity in human skin cells.

The current study has several limitations. Though the skin cell culture was designed to model the disease biology of chronic inflammation, the *in vitro* study results cannot be directly translated to the more complex human system. The impact of LEO on gene expression was analyzed after short-term intervention. How LEO impacts global gene expression over a longer term remains elusive. Nevertheless, based on the protein and gene expression data, this study provides important evidence of the biological effect of LEO on human skin cells and will likely stimulate further research into LEO's mechanisms of action.

## 4 Conclusions

To our knowledge, this is the first study to evaluate the anti-inflammatory activity of LEO, which has citral as its main component, on pre-inflamed human dermal fibroblasts. LEO showed significant inhibition of VCAM-1, IP-10, I-TAC, and MIG production. It also significantly decreased collagen-I and III, EGFR, M-CSF, and PAI-1. Genome-wide gene expression analysis demonstrated robust and diverse effects of LEO. Many of the most impacted genes and pathways are critically involved in inflammation processes, supporting the anti-inflammatory property of LEO. LEO is likely to be a good therapeutic candidate for treating skin inflammation.

## Conflicts of interest

X.H. and T.P. are employees of dōTERRA, where the study agent LEO was manufactured.

## Footnotes

1 LEO, Lemongrass essential oil.
