# Supplementary material for: Lemongrass (Cymbopogon flexuosus) essential oil demonstrated anti-inflammatory effect in pre-inflamed human dermal fibroblasts
Source: Biochim Open. 2017 Mar 21;4:107–11. doi: 10.1016/j.biopen.2017.03.004 (PMC5801909; doi:10.1016/j.biopen.2017.03.004)

Table S1. Glossary of biomarkers of the human dermal fibroblast system HDF3CGF used in the study

| **Readout** | **Description** |
| --- | --- |
| **CCL2/MCP-1** | MCP-1 system is a chemokine that mediates recruitment of monocytes and T cells into sites of inflammation. MCP-1 is categorized as an inflammation-related activity in the HDF3CGF system modeling Th1 inflammation involved in wound healing and matrix remodeling. |
| **CD106/VCAM-1** | VCAM-1 is a cell adhesion molecule that mediates adhesion of monocytes and T cells to endothelial cells. VCAM-1 is categorized as an inflammation-related activity. |
| **CD54/ICAM-1** | ICAM-1 is a cell adhesion molecule that mediates leukocyte-endothelial cell adhesion and leukocyte recruitment. ICAM-1 is categorized as an inflammation-related activity. |
| **Collagen I** | Collagen I is involved in tissue remodeling and fibrosis, and is the most common fibrillar collagen that is found in skin, bone, tendons and other connective tissues. Collagen I is categorized as a tissue remodeling-related activity. |
| **Collagen III** | Collagen III is an extracellular matrix protein and fibrillar collagen found in extensible connective tissues (skin, lung and vascular system) and is involved in cell adhesion, cell migration, tissue remodeling. Collagen III is categorized as a tissue remodeling-related activity. |
| **CXCL10/IP-10** | IP-10 is a chemokine that mediates T cell, monocyte and dendritic cell chemotaxis. IP-10 is categorized as an inflammation-related activity. |
| **CXCL11/I-TAC** | I-TAC is a chemokine that mediates T cell and monocyte chemotaxis. I-TAC is categorized as an inflammation-related activity. |
| **CXCL8/IL-8** | IL-8 is a chemokine that mediates neutrophil recruitment into acute inflammatory sites. IL-8 is categorized as an inflammation-related activity. |
| **CXCL9/MIG** | MIG is a chemokine that mediates T cell recruitment. MIG is categorized as an inflammation-related activity. |
| **EGFR** | EGFR is a cell surface receptor for epidermal growth factor involved in cell proliferation during development as well as tumor growth. EGFR is involved in Epithelial cell proliferation, epithelial cell differentiation keratinocyte proliferation, tissue remodeling. EGFR is categorized as a tissue remodeling-related activity. |
| **M-CSF** | M-CSF is a secreted and cell surface cytokine that mediates macrophage differentiation. M-CSF is categorized as an immune modulation-related activity. |
| **MMP-1** | MMP-1 is an interstitial collagenase that degrades collagens I, II and III and is involved in the process of tissue remodeling. MMP-1 is categorized as a tissue remodeling-related activity. |
| **PAI-I** | PAI-I is a serine proteinase inhibitor and inhibitor of tissue plasminogen activator (tPA) and urokinase (uPA) and is involved in tissue remodeling and fibrinolysis. PAI-I is categorized as a tissue remodeling-related activity. |
| **Proliferation_72hr** | Proliferation_72hr in the HDF3CGF system is a measure of dermal fibroblast proliferation which is important to the process of wound healing and fibrosis. |
| **SRB** | SRB is a measure of the total protein content of dermal fibroblasts. Cell viability of adherent cells is measured by Sulforhodamine B (SRB) staining, a method that determines cell density by measuring total protein content of test wells. |
| **TIMP-1** | TIMP-1 is a tissue inhibitor of matrix metalloprotease-7 (MMP-7) and other MMPs, and is involved in tissue remodeling, angiogenesis and fibrosis. TIMP-1 is categorized as a tissue remodeling-related activity. |
| **TIMP-2** | TIMP-2 is a tissue inhibitor of matrix metalloproteases and is involved in tissue remodeling, angiogenesis and fibrosis. TIMP-2 is categorized as a tissue remodeling-related activity. |

Table S2. The 200 most impacted genes by lemongrass essential oil (LEO, 0.0012% v/v)

| **Illumina Gene ID** | **Fold Change in Log_2_ form** | **Definition** |
| --- | --- | --- |
| ADH1A | 4.26 | Homo sapiens alcohol dehydrogenase 1A (class I), alpha polypeptide (ADH1A), mRNA. |
| HSPE1 | 2.81 | Homo sapiens heat shock 10kDa protein 1 (chaperonin 10) (HSPE1), mRNA. |
| COL3A1 | 2.76 | Homo sapiens collagen, type III, alpha 1 (COL3A1), mRNA. |
| AKR1C4 | 2.57 | Homo sapiens aldo-keto reductase family 1, member C4 (chlordecone reductase; 3-alpha hydroxysteroid dehydrogenase, type I; dihydrodiol dehydrogenase 4) (AKR1C4), mRNA. |
| AKR1C2 | 2.47 | Homo sapiens aldo-keto reductase family 1, member C2 (dihydrodiol dehydrogenase 2; bile acid binding protein; 3-alpha hydroxysteroid dehydrogenase, type III) (AKR1C2), transcript variant 1, mRNA. XM_943424 XM_943425 XM_943427 |
| ALDH1A1 | 2.29 | Homo sapiens aldehyde dehydrogenase 1 family, member A1 (ALDH1A1), mRNA. |
| ALDH1A1 | 2.26 | Homo sapiens aldehyde dehydrogenase 1 family, member A1 (ALDH1A1), mRNA. |
| CXCL12 | 2.22 | Homo sapiens chemokine (C-X-C motif) ligand 12 (stromal cell-derived factor 1) (CXCL12), transcript variant 1, mRNA. |
| TFRC | 2.21 | Homo sapiens transferrin receptor (p90, CD71) (TFRC), mRNA. |
| CLDN11 | 2.13 | Homo sapiens claudin 11 (oligodendrocyte transmembrane protein) (CLDN11), mRNA. |
| COL1A1 | 2.11 | Homo sapiens collagen, type I, alpha 1 (COL1A1), mRNA. |
| LOC441743 | 2.10 | Homo sapiens similar to C367G8.3 (novel protein similar to RPL23A (60S ribosomal protein L23A)) (LOC441743), mRNA. |
| TSPAN13 | 2.09 | Homo sapiens tetraspanin 13 (TSPAN13), mRNA. |
| PDGFD | 2.04 | Homo sapiens platelet derived growth factor D (PDGFD), transcript variant 2, mRNA. |
| TNKS2 | 2.03 | Homo sapiens tankyrase, TRF1-interacting ankyrin-related ADP-ribose polymerase 2 (TNKS2), mRNA. |
| CYP26B1 | 2.02 | Homo sapiens cytochrome P450, family 26, subfamily B, polypeptide 1 (CYP26B1), mRNA. |
| TDO2 | 1.97 | Homo sapiens tryptophan 2,3-dioxygenase (TDO2), mRNA. |
| SLC7A11 | 1.95 | Homo sapiens solute carrier family 7, (cationic amino acid transporter, y+ system) member 11 (SLC7A11), mRNA. |
| OSR2 | 1.89 | PREDICTED: Homo sapiens odd-skipped related 2 (Drosophila) (OSR2), mRNA. |
| HNRNPA1 | 1.85 | Homo sapiens heterogeneous nuclear ribonucleoprotein A1 (HNRNPA1), transcript variant 2, mRNA. |
| CEPT1 | 1.84 | Homo sapiens choline/ethanolamine phosphotransferase 1 (CEPT1), transcript variant 1, mRNA. |
| CXCL12 | 1.83 | Homo sapiens chemokine (C-X-C motif) ligand 12 (stromal cell-derived factor 1) (CXCL12), transcript variant 2, mRNA. |
| TSPAN13 | 1.82 | Homo sapiens tetraspanin 13 (TSPAN13), mRNA. |
| ANGPTL2 | 1.82 | Homo sapiens angiopoietin-like 2 (ANGPTL2), mRNA. |
| TSPAN1 | 1.77 | Homo sapiens tetraspanin 1 (TSPAN1), mRNA. |
| HNRPH1 | 1.73 | Homo sapiens heterogeneous nuclear ribonucleoprotein H1 (H) (HNRPH1), mRNA. |
| ANGPTL4 | 1.70 | Homo sapiens angiopoietin-like 4 (ANGPTL4), transcript variant 1, mRNA. |
| RPS7 | 1.69 | Homo sapiens ribosomal protein S7 (RPS7), mRNA. |
| FABP5 | 1.68 | Homo sapiens fatty acid binding protein 5 (psoriasis-associated) (FABP5), mRNA. |
| HS.544245 | 1.68 | Homo sapiens clone FBA2 Cri-du-chat region mRNA |
| C6ORF66 | 1.68 | Homo sapiens chromosome 6 open reading frame 66 (C6orf66), mRNA. |
| RGS2 | 1.66 | Homo sapiens regulator of G-protein signalling 2, 24kDa (RGS2), mRNA. |
| RPS7 | 1.66 | Homo sapiens ribosomal protein S7 (RPS7), mRNA. |
| OAZ2 | 1.65 | Homo sapiens ornithine decarboxylase antizyme 2 (OAZ2), mRNA. |
| RPL29 | 1.63 | Homo sapiens ribosomal protein L29 (RPL29), mRNA. |
| RPLP1 | 1.62 | Homo sapiens ribosomal protein, large, P1 (RPLP1), transcript variant 1, mRNA. |
| RPS15 | 1.62 | Homo sapiens ribosomal protein S15 (RPS15), mRNA. |
| GPR182 | 1.61 | Homo sapiens G protein-coupled receptor 182 (GPR182), mRNA. |
| FABP4 | 1.61 | Homo sapiens fatty acid binding protein 4, adipocyte (FABP4), mRNA. |
| HS.527671 | 1.61 | UI-CF-FN0-age-p-21-18-UI.r18 UI-CF-FN0 Homo sapiens cDNA clone UI-CF-FN0-age-p-21-18-UI 5, mRNA sequence |
| ODC1 | 1.60 | Homo sapiens ornithine decarboxylase 1 (ODC1), mRNA. |
| C4ORF18 | 1.60 | Homo sapiens chromosome 4 open reading frame 18 (C4orf18), transcript variant 2, mRNA. |
| ASNA1 | 1.60 | Homo sapiens arsA arsenite transporter, ATP-binding, homolog 1 (bacterial) (ASNA1), mRNA. |
| LOC643438 | 1.60 | PREDICTED: Homo sapiens misc_RNA (LOC643438), miscRNA. |
| RPL29 | 1.60 | Homo sapiens ribosomal protein L29 (RPL29), mRNA. |
| EEF1B2 | 1.59 | Homo sapiens eukaryotic translation elongation factor 1 beta 2 (EEF1B2), transcript variant 1, mRNA. |
| CTR9 | 1.58 | Homo sapiens Ctr9, Paf1/RNA polymerase II complex component, homolog (S. cerevisiae) (CTR9), mRNA. |
| GPC1 | 1.58 | Homo sapiens glypican 1 (GPC1), mRNA. |
| FAM59A | 1.58 | Homo sapiens family with sequence similarity 59, member A (FAM59A), mRNA. |
| CCDC19 | 1.58 | Homo sapiens coiled-coil domain containing 19 (CCDC19), mRNA. |
| LOC346887 | 1.58 | PREDICTED: Homo sapiens similar to solute carrier family 16 (monocarboxylic acid transporters), member 14 (LOC346887), mRNA. |
| LOC100130562 | 1.57 | PREDICTED: Homo sapiens hypothetical protein LOC100130562, transcript variant 1 (LOC100130562), mRNA. |
| OSR1 | 1.57 | Homo sapiens odd-skipped related 1 (Drosophila) (OSR1), mRNA. |
| LOC606724 | 1.56 | Homo sapiens coronin, actin binding protein, 1A pseudogene (LOC606724), non-coding RNA. |
| GLDN | 1.55 | Homo sapiens gliomedin (GLDN), mRNA. |
| LOC645231 | 1.55 | PREDICTED: Homo sapiens misc_RNA (LOC645231), miscRNA. |
| LOC387934 | 1.55 | PREDICTED: Homo sapiens similar to Fatty acid-binding protein, epidermal (E-FABP) (Psoriasis-associated fatty acid-binding protein homolog) (PA-FABP) (LOC387934), mRNA. |
| AGAP3 | 1.55 | Homo sapiens ArfGAP with GTPase domain, ankyrin repeat and PH domain 3 (AGAP3), transcript variant 2, mRNA. |
| NGRN | 1.55 | Homo sapiens neugrin, neurite outgrowth associated (NGRN), transcript variant 1, mRNA. |
| TDO2 | 1.54 | Homo sapiens tryptophan 2,3-dioxygenase (TDO2), mRNA. |
| LOC644496 | 1.54 | PREDICTED: Homo sapiens misc_RNA (LOC644496), miscRNA. |
| LOC401357 | 1.54 | Homo sapiens hypothetical LOC401357 (LOC401357), mRNA. |
| CMBL | 1.54 | Homo sapiens carboxymethylenebutenolidase homolog (Pseudomonas) (CMBL), mRNA. |
| C21ORF86 | 1.54 | Homo sapiens chromosome 21 open reading frame 86 (C21orf86), mRNA. |
| RPS17 | 1.54 | Homo sapiens ribosomal protein S17 (RPS17), mRNA. |
| ACSL5 | -1.53 | Homo sapiens acyl-CoA synthetase long-chain family member 5 (ACSL5), transcript variant 2, mRNA. |
| CNBD1 | -1.53 | Homo sapiens cyclic nucleotide binding domain containing 1 (CNBD1), mRNA. |
| RHOU | -1.53 | Homo sapiens ras homolog gene family, member U (RHOU), mRNA. |
| PPARD | -1.53 | Homo sapiens peroxisome proliferative activated receptor, delta (PPARD), transcript variant 1, mRNA. |
| PDE4D | -1.54 | Homo sapiens phosphodiesterase 4D, cAMP-specific (phosphodiesterase E3 dunce homolog, Drosophila) (PDE4D), mRNA. |
| DES | -1.54 | Homo sapiens desmin (DES), mRNA. |
| NOD2 | -1.54 | Homo sapiens nucleotide-binding oligomerization domain containing 2 (NOD2), mRNA. |
| MASP1 | -1.54 | Homo sapiens mannan-binding lectin serine peptidase 1 (C4/C2 activating component of Ra-reactive factor) (MASP1), transcript variant 2, mRNA. |
| ADAMDEC1 | -1.54 | Homo sapiens ADAM-like, decysin 1 (ADAMDEC1), mRNA. |
| TXNDC11 | -1.54 | Homo sapiens thioredoxin domain containing 11 (TXNDC11), mRNA. |
| CDKN3 | -1.54 | Homo sapiens cyclin-dependent kinase inhibitor 3 (CDK2-associated dual specificity phosphatase) (CDKN3), mRNA. |
| NUSAP1 | -1.54 | Homo sapiens nucleolar and spindle associated protein 1 (NUSAP1), transcript variant 2, mRNA. |
| SDC4 | -1.54 | Homo sapiens syndecan 4 (SDC4), mRNA. |
| RASL11B | -1.54 | Homo sapiens RAS-like, family 11, member B (RASL11B), mRNA. |
| LAMB3 | -1.54 | Homo sapiens laminin, beta 3 (LAMB3), transcript variant 1, mRNA. |
| EPHA3 | -1.54 | Homo sapiens EPH receptor A3 (EPHA3), transcript variant 1, mRNA. |
| WNK4 | -1.54 | Homo sapiens WNK lysine deficient protein kinase 4 (WNK4), mRNA. |
| STX11 | -1.55 | Homo sapiens syntaxin 11 (STX11), mRNA. |
| SCARA3 | -1.55 | Homo sapiens scavenger receptor class A, member 3 (SCARA3), transcript variant 2, mRNA. |
| SLC43A3 | -1.55 | Homo sapiens solute carrier family 43, member 3 (SLC43A3), mRNA. |
| INSIG2 | -1.55 | Homo sapiens insulin induced gene 2 (INSIG2), mRNA. |
| FBLN7 | -1.55 | Homo sapiens fibulin 7 (FBLN7), mRNA. |
| METRNL | -1.55 | Homo sapiens meteorin, glial cell differentiation regulator-like (METRNL), mRNA. |
| HS.582025 | -1.55 | ov47h04.x1 Soares_testis_NHT Homo sapiens cDNA clone IMAGE:1640503 3, mRNA sequence |
| TOP2A | -1.55 | Homo sapiens topoisomerase (DNA) II alpha 170kDa (TOP2A), mRNA. |
| SCARA3 | -1.56 | Homo sapiens scavenger receptor class A, member 3 (SCARA3), transcript variant 1, mRNA. |
| MMP9 | -1.56 | Homo sapiens matrix metallopeptidase 9 (gelatinase B, 92kDa gelatinase, 92kDa type IV collagenase) (MMP9), mRNA. |
| TP53INP2 | -1.56 | Homo sapiens tumor protein p53 inducible nuclear protein 2 (TP53INP2), mRNA. |
| GIMAP4 | -1.56 | Homo sapiens GTPase, IMAP family member 4 (GIMAP4), mRNA. |
| PI3 | -1.56 | Homo sapiens peptidase inhibitor 3, skin-derived (SKALP) (PI3), mRNA. |
| TNFAIP6 | -1.56 | Homo sapiens tumor necrosis factor, alpha-induced protein 6 (TNFAIP6), mRNA. |
| RNF19B | -1.56 | Homo sapiens ring finger protein 19B (RNF19B), mRNA. |
| PPP1R12B | -1.56 | Homo sapiens protein phosphatase 1, regulatory (inhibitor) subunit 12B (PPP1R12B), transcript variant 3, mRNA. |
| UGCG | -1.56 | Homo sapiens UDP-glucose ceramide glucosyltransferase (UGCG), mRNA. |
| BNIP3 | -1.57 | Homo sapiens BCL2/adenovirus E1B 19kDa interacting protein 3 (BNIP3), nuclear gene encoding mitochondrial protein, mRNA. |
| GRLF1 | -1.57 | Homo sapiens glucocorticoid receptor DNA binding factor 1 (GRLF1), mRNA. |
| DDX60 | -1.57 | Homo sapiens DEAD (Asp-Glu-Ala-Asp) box polypeptide 60 (DDX60), mRNA. |
| FGF11 | -1.58 | Homo sapiens fibroblast growth factor 11 (FGF11), mRNA. |
| LOC731007 | -1.58 | PREDICTED: Homo sapiens similar to Adenylate kinase isoenzyme 4, mitochondrial (Adenylate kinase 3-like 1) (ATP-AMP transphosphorylase) (LOC731007), mRNA. |
| LOC100134140 | -1.58 | PREDICTED: Homo sapiens similar to LHPE306, transcript variant 3 (LOC100134140), mRNA. |
| CDH13 | -1.59 | Homo sapiens cadherin 13, H-cadherin (heart) (CDH13), mRNA. |
| SLC39A8 | -1.59 | Homo sapiens solute carrier family 39 (zinc transporter), member 8 (SLC39A8), transcript variant 1, mRNA. |
| CD38 | -1.59 | Homo sapiens CD38 molecule (CD38), mRNA. |
| NTN1 | -1.59 | Homo sapiens netrin 1 (NTN1), mRNA. |
| CMAH | -1.59 | Homo sapiens cytidine monophosphate-N-acetylneuraminic acid hydroxylase (CMP-N-acetylneuraminate monooxygenase) pseudogene (CMAH), transcript variant 1, non-coding RNA. |
| LOC649841 | -1.59 | PREDICTED: Homo sapiens similar to protein immuno-reactive with anti-PTH polyclonal antibodies (LOC649841), mRNA. |
| STAT4 | -1.60 | Homo sapiens signal transducer and activator of transcription 4 (STAT4), mRNA. |
| CXCR3 | -1.60 | Homo sapiens chemokine (C-X-C motif) receptor 3 (CXCR3), transcript variant A, mRNA. |
| PIM1 | -1.60 | Homo sapiens pim-1 oncogene (PIM1), mRNA. |
| MT1G | -1.60 | Homo sapiens metallothionein 1G (MT1G), mRNA. |
| HLA-DRB6 | -1.60 | Homo sapiens major histocompatibility complex, class II, DR beta 6 (pseudogene) (HLA-DRB6), non-coding RNA. |
| CFB | -1.61 | Homo sapiens complement factor B (CFB), mRNA. |
| LYN | -1.61 | Homo sapiens v-yes-1 Yamaguchi sarcoma viral related oncogene homolog (LYN), mRNA. |
| RARRES1 | -1.62 | Homo sapiens retinoic acid receptor responder (tazarotene induced) 1 (RARRES1), transcript variant 1, mRNA. |
| GNA15 | -1.62 | Homo sapiens guanine nucleotide binding protein (G protein), alpha 15 (Gq class) (GNA15), mRNA. |
| GRAMD3 | -1.63 | Homo sapiens GRAM domain containing 3 (GRAMD3), mRNA. |
| STC2 | -1.63 | Homo sapiens stanniocalcin 2 (STC2), mRNA. |
| TLE1 | -1.64 | Homo sapiens transducin-like enhancer of split 1 (E(sp1) homolog, Drosophila) (TLE1), mRNA. |
| ANLN | -1.64 | Homo sapiens anillin, actin binding protein (ANLN), mRNA. |
| TEX11 | -1.64 | Homo sapiens testis expressed 11 (TEX11), transcript variant 1, mRNA. |
| HLA-DPA1 | -1.65 | Homo sapiens major histocompatibility complex, class II, DP alpha 1 (HLA-DPA1), mRNA. |
| SYNC1 | -1.65 | Homo sapiens syncoilin, intermediate filament 1 (SYNC1), mRNA. |
| MUC1 | -1.65 | Homo sapiens mucin 1, cell surface associated (MUC1), transcript variant 5, mRNA. |
| IGFBP3 | -1.65 | Homo sapiens insulin-like growth factor binding protein 3 (IGFBP3), transcript variant 2, mRNA. |
| HS.565908 | -1.65 | UI-E-EO1-aiv-l-12-0-UI.s1 UI-E-EO1 Homo sapiens cDNA clone UI-E-EO1-aiv-l-12-0-UI 3, mRNA sequence |
| LOC284276 | -1.66 | Homo sapiens hypothetical LOC284276 (LOC284276), non-coding RNA. |
| SIRPA | -1.66 | Homo sapiens signal-regulatory protein alpha (SIRPA), transcript variant 3, mRNA. |
| FAM20A | -1.66 | Homo sapiens family with sequence similarity 20, member A (FAM20A), mRNA. |
| HLA-DRB5 | -1.66 | Homo sapiens major histocompatibility complex, class II, DR beta 5 (HLA-DRB5), mRNA. |
| CFH | -1.67 | Homo sapiens complement factor H (CFH), transcript variant 1, mRNA. |
| VCAM1 | -1.67 | Homo sapiens vascular cell adhesion molecule 1 (VCAM1), transcript variant 1, mRNA. |
| NCCRP1 | -1.67 | Homo sapiens non-specific cytotoxic cell receptor protein 1 homolog (zebrafish) (NCCRP1), mRNA. |
| SRGN | -1.68 | Homo sapiens serglycin (SRGN), mRNA. |
| SEPT4 | -1.68 | Homo sapiens septin 4 (SEPT4), transcript variant 1, mRNA. |
| CYB5A | -1.68 | Homo sapiens cytochrome b5 type A (microsomal) (CYB5A), transcript variant 2, mRNA. |
| METTL7A | -1.68 | Homo sapiens methyltransferase like 7A (METTL7A), mRNA. |
| TMEM132A | -1.68 | Homo sapiens transmembrane protein 132A (TMEM132A), transcript variant 2, mRNA. |
| LOC100133797 | -1.68 | PREDICTED: Homo sapiens hypothetical protein LOC100133797 (LOC100133797), mRNA. |
| SRGN | -1.70 | Homo sapiens serglycin (SRGN), mRNA. |
| BNIP3L | -1.70 | Homo sapiens BCL2/adenovirus E1B 19kDa interacting protein 3-like (BNIP3L), mRNA. |
| CXCR7 | -1.70 | Homo sapiens chemokine (C-X-C motif) receptor 7 (CXCR7), transcript variant 1, mRNA. |
| HECW2 | -1.71 | Homo sapiens HECT, C2 and WW domain containing E3 ubiquitin protein ligase 2 (HECW2), mRNA. |
| LOC649143 | -1.72 | PREDICTED: Homo sapiens similar to HLA class II histocompatibility antigen, DRB1-9 beta chain precursor (MHC class I antigen DRB1*9) (DR-9) (DR9), transcript variant 2 (LOC649143), mRNA. |
| NHS | -1.73 | Homo sapiens Nance-Horan syndrome (congenital cataracts and dental anomalies) (NHS), transcript variant 1, mRNA. |
| HS.546379 | -1.74 | Homo sapiens T-cell receptor alpha chain (TCRA) mRNA (HLA-A1, 24; B7, 8; DR 1, 3), complete cds |
| IFIT2 | -1.75 | Homo sapiens interferon-induced protein with tetratricopeptide repeats 2 (IFIT2), mRNA. |
| SLC39A8 | -1.75 | Homo sapiens solute carrier family 39 (zinc transporter), member 8 (SLC39A8), transcript variant 1, mRNA. |
| ENO2 | -1.75 | Homo sapiens enolase 2 (gamma, neuronal) (ENO2), mRNA. |
| CLDN7 | -1.77 | Homo sapiens claudin 7 (CLDN7), mRNA. |
| RARRES1 | -1.77 | Homo sapiens retinoic acid receptor responder (tazarotene induced) 1 (RARRES1), transcript variant 2, mRNA. |
| TMEM45A | -1.77 | Homo sapiens transmembrane protein 45A (TMEM45A), mRNA. |
| H2AFY2 | -1.77 | Homo sapiens H2A histone family, member Y2 (H2AFY2), mRNA. |
| HLA-DRB1 | -1.79 | Homo sapiens major histocompatibility complex, class II, DR beta 1 (HLA-DRB1), mRNA. |
| PLAT | -1.79 | Homo sapiens plasminogen activator, tissue (PLAT), transcript variant 1, mRNA. |
| ABI3BP | -1.80 | Homo sapiens ABI gene family, member 3 (NESH) binding protein (ABI3BP), mRNA. |
| PSTPIP2 | -1.82 | Homo sapiens proline-serine-threonine phosphatase interacting protein 2 (PSTPIP2), mRNA. |
| HLA-DRA | -1.82 | Homo sapiens major histocompatibility complex, class II, DR alpha (HLA-DRA), mRNA. |
| NGF | -1.82 | Homo sapiens nerve growth factor (beta polypeptide) (NGF), mRNA. |
| IGFBP7 | -1.82 | Homo sapiens insulin-like growth factor binding protein 7 (IGFBP7), mRNA. |
| HLA-DRA | -1.85 | Homo sapiens major histocompatibility complex, class II, DR alpha (HLA-DRA), mRNA. |
| MT1M | -1.86 | Homo sapiens metallothionein 1M (MT1M), mRNA. |
| HS.10862 | -1.91 | Homo sapiens cDNA: FLJ23313 fis, clone HEP11919 |
| AK3L1 | -1.93 | Homo sapiens adenylate kinase 3-like 1 (AK3L1), nuclear gene encoding mitochondrial protein, transcript variant 7, mRNA. |
| DNER | -1.94 | Homo sapiens delta/notch-like EGF repeat containing (DNER), mRNA. |
| HSD11B1 | -1.94 | Homo sapiens hydroxysteroid (11-beta) dehydrogenase 1 (HSD11B1), transcript variant 2, mRNA. |
| MMP12 | -1.96 | Homo sapiens matrix metallopeptidase 12 (macrophage elastase) (MMP12), mRNA. |
| CD74 | -1.96 | Homo sapiens CD74 molecule, major histocompatibility complex, class II invariant chain (CD74), transcript variant 2, mRNA. |
| SEPT4 | -1.97 | Homo sapiens septin 4 (SEPT4), transcript variant 3, mRNA. |
| SAA1 | -1.98 | Homo sapiens serum amyloid A1 (SAA1), transcript variant 2, mRNA. |
| IL4I1 | -1.98 | Homo sapiens interleukin 4 induced 1 (IL4I1), transcript variant 2, mRNA. |
| HSD11B1 | -1.98 | Homo sapiens hydroxysteroid (11-beta) dehydrogenase 1 (HSD11B1), transcript variant 2, mRNA. |
| CCL5 | -1.99 | Homo sapiens chemokine (C-C motif) ligand 5 (CCL5), mRNA. |
| MUC1 | -1.99 | Homo sapiens mucin 1, cell surface associated (MUC1), transcript variant 6, mRNA. |
| VCAM1 | -1.99 | Homo sapiens vascular cell adhesion molecule 1 (VCAM1), transcript variant 1, mRNA. |
| MT1F | -1.99 | Homo sapiens metallothionein 1F (MT1F), mRNA. |
| SEPT4 | -2.00 | Homo sapiens septin 4 (SEPT4), transcript variant 2, mRNA. |
| MT3 | -2.00 | Homo sapiens metallothionein 3 (MT3), mRNA. |
| STEAP4 | -2.01 | Homo sapiens STEAP family member 4 (STEAP4), mRNA. |
| UBD | -2.04 | Homo sapiens ubiquitin D (UBD), mRNA. |
| HLA-DRB4 | -2.05 | Homo sapiens major histocompatibility complex, class II, DR beta 4 (HLA-DRB4), mRNA. |
| HSD11B1 | -2.07 | Homo sapiens hydroxysteroid (11-beta) dehydrogenase 1 (HSD11B1), transcript variant 1, mRNA. |
| HIST1H4C | -2.09 | Homo sapiens histone cluster 1, H4c (HIST1H4C), mRNA. |
| AK3L1 | -2.09 | Homo sapiens adenylate kinase 3-like 1 (AK3L1), nuclear gene encoding mitochondrial protein, transcript variant 7, mRNA. |
| LIPG | -2.11 | Homo sapiens lipase, endothelial (LIPG), mRNA. |
| ASCL2 | -2.18 | Homo sapiens achaete-scute complex homolog 2 (Drosophila) (ASCL2), mRNA. |
| LOC730415 | -2.22 | PREDICTED: Homo sapiens hypothetical LOC730415, transcript variant 2 (LOC730415), mRNA. |
| MMP12 | -2.24 | Homo sapiens matrix metallopeptidase 12 (macrophage elastase) (MMP12), mRNA. |
| HAS3 | -2.30 | Homo sapiens hyaluronan synthase 3 (HAS3), transcript variant 1, mRNA. |
| CX3CL1 | -2.34 | Homo sapiens chemokine (C-X3-C motif) ligand 1 (CX3CL1), mRNA. |
| CD74 | -2.34 | Homo sapiens CD74 molecule, major histocompatibility complex, class II invariant chain (CD74), transcript variant 1, mRNA. |
| MT1JP | -2.45 | Homo sapiens metallothionein 1J (pseudogene) (MT1JP), mRNA. |
| MT1H | -2.53 | Homo sapiens metallothionein 1H (MT1H), mRNA. |
| CCL5 | -2.72 | Homo sapiens chemokine (C-C motif) ligand 5 (CCL5), mRNA. |
| MYH11 | -2.93 | Homo sapiens myosin, heavy chain 11, smooth muscle (MYH11), transcript variant SM1A, mRNA. |
| SLC2A5 | -3.04 | Homo sapiens solute carrier family 2 (facilitated glucose/fructose transporter), member 5 (SLC2A5), mRNA. |

Table S3. Top 20 genes regulated by lemongrass essential oil (LEO, 0.0012% v/v) in the canonical hepatic fibrosis/hepatic stellate cell activation pathway. Fold change over vehicle was shown in log_2_ ratio form.


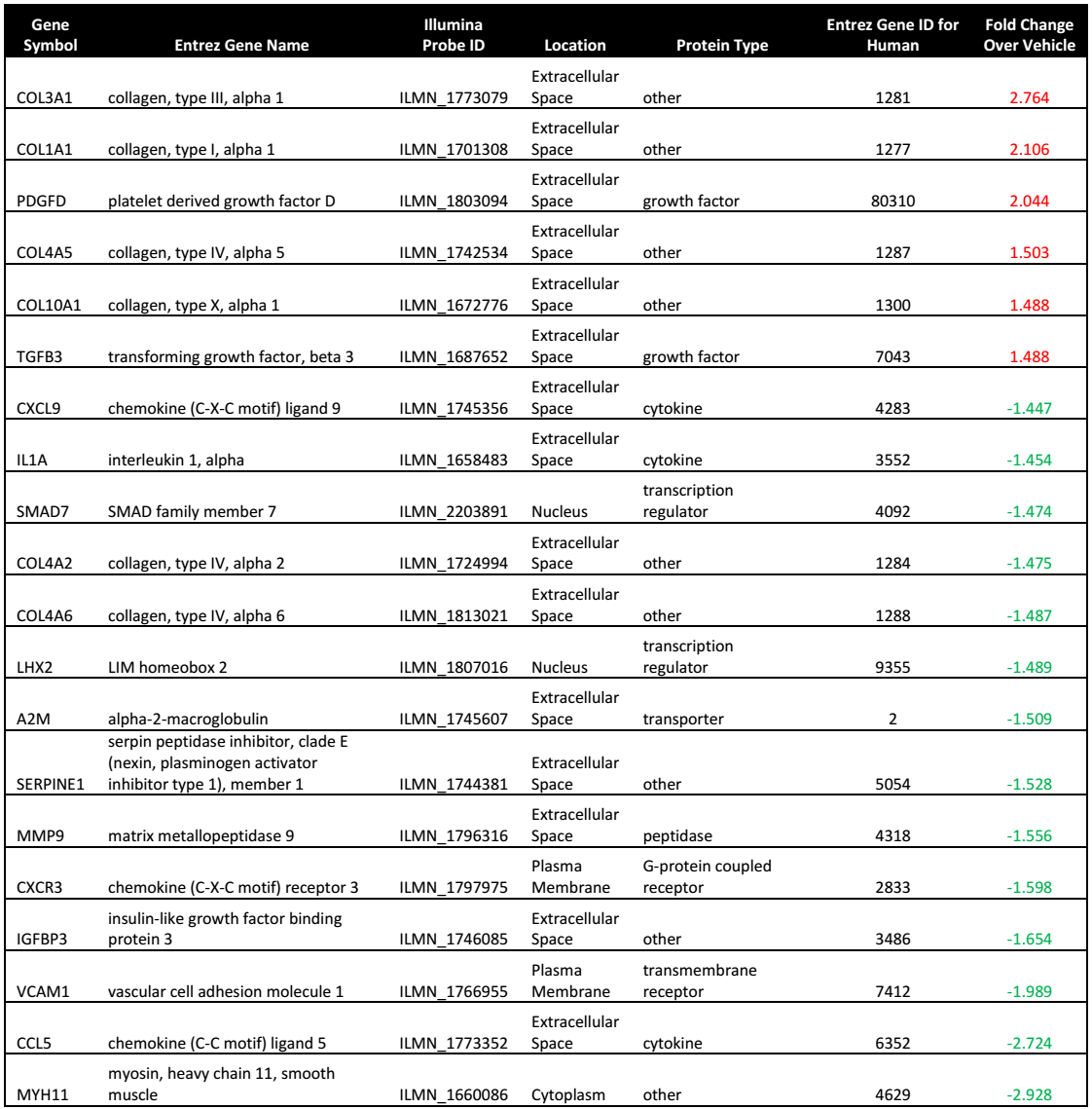


Table S4. Top 17 genes regulated by lemongrass essential oil (LEO, 0.0012% v/v) in the canonical antigen presentation pathway. Fold change over vehicle was shown in log_2_ ratio form.


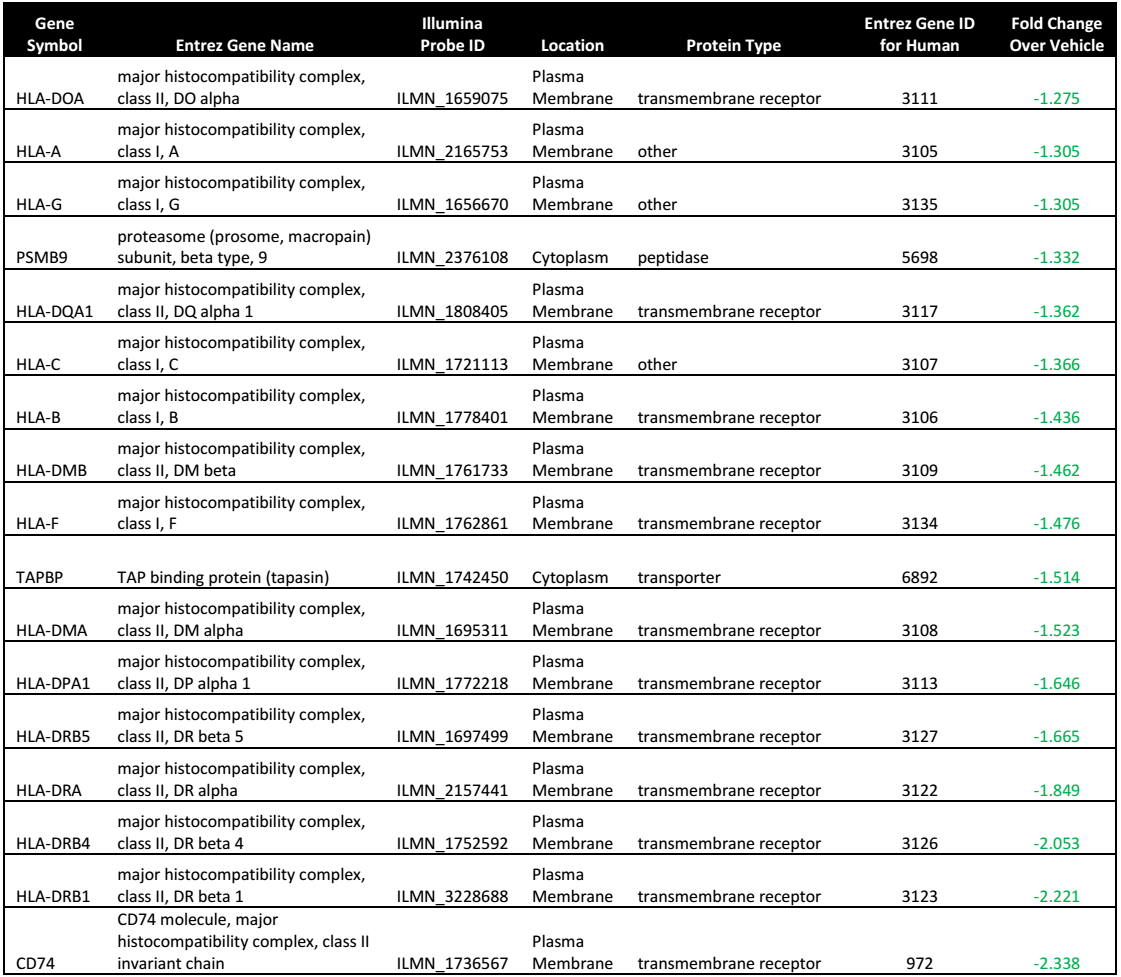


Table S5. Top 17 genes regulated by lemongrass essential oil (LEO, 0.0012% v/v) in the canonical graft-versus-host disease signaling pathway. Fold change over vehicle was shown in log_2_ ratio form.


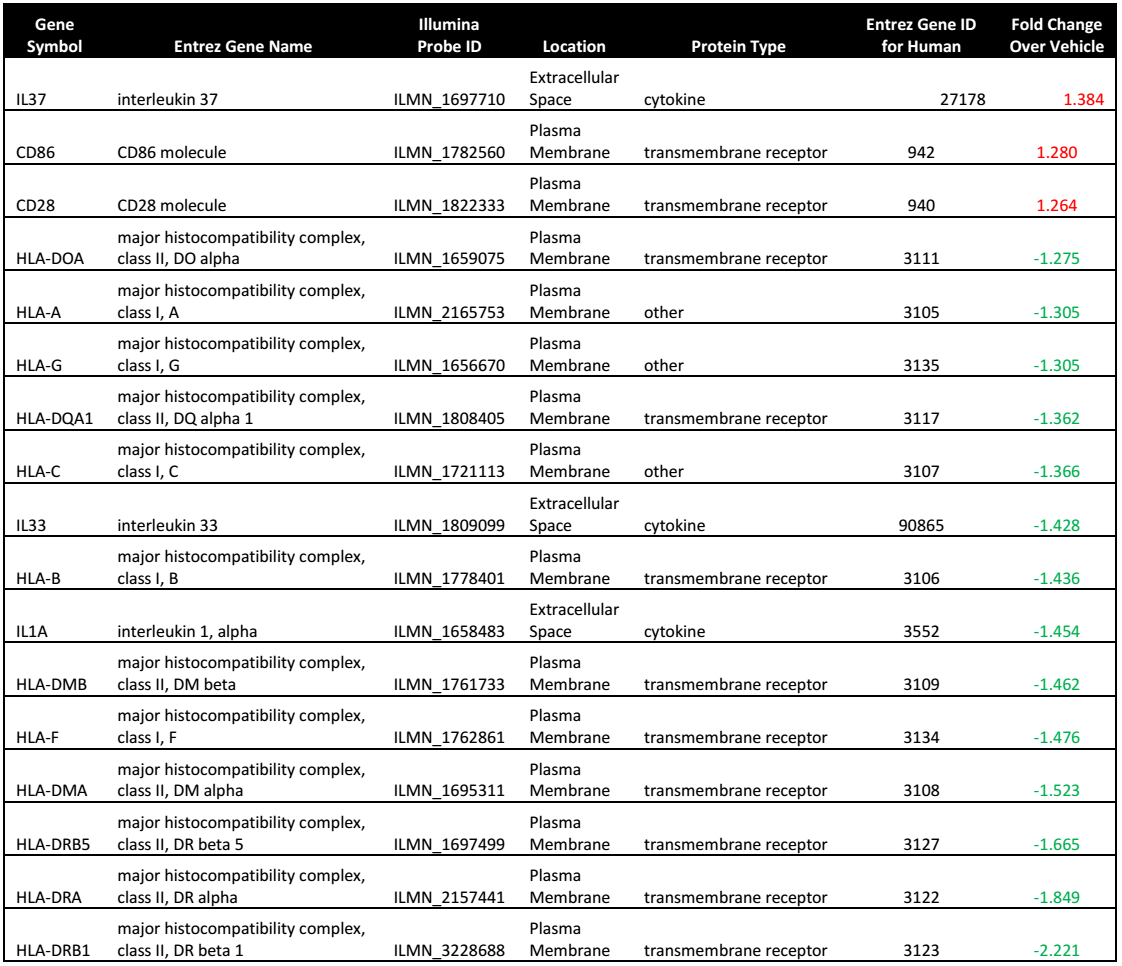


Table S6. Top 20 genes regulated by lemongrass essential oil (LEO, 0.0012% v/v) in the canonical dendritic cell maturation pathway. Fold change over vehicle was shown in log_2_ ratio form.
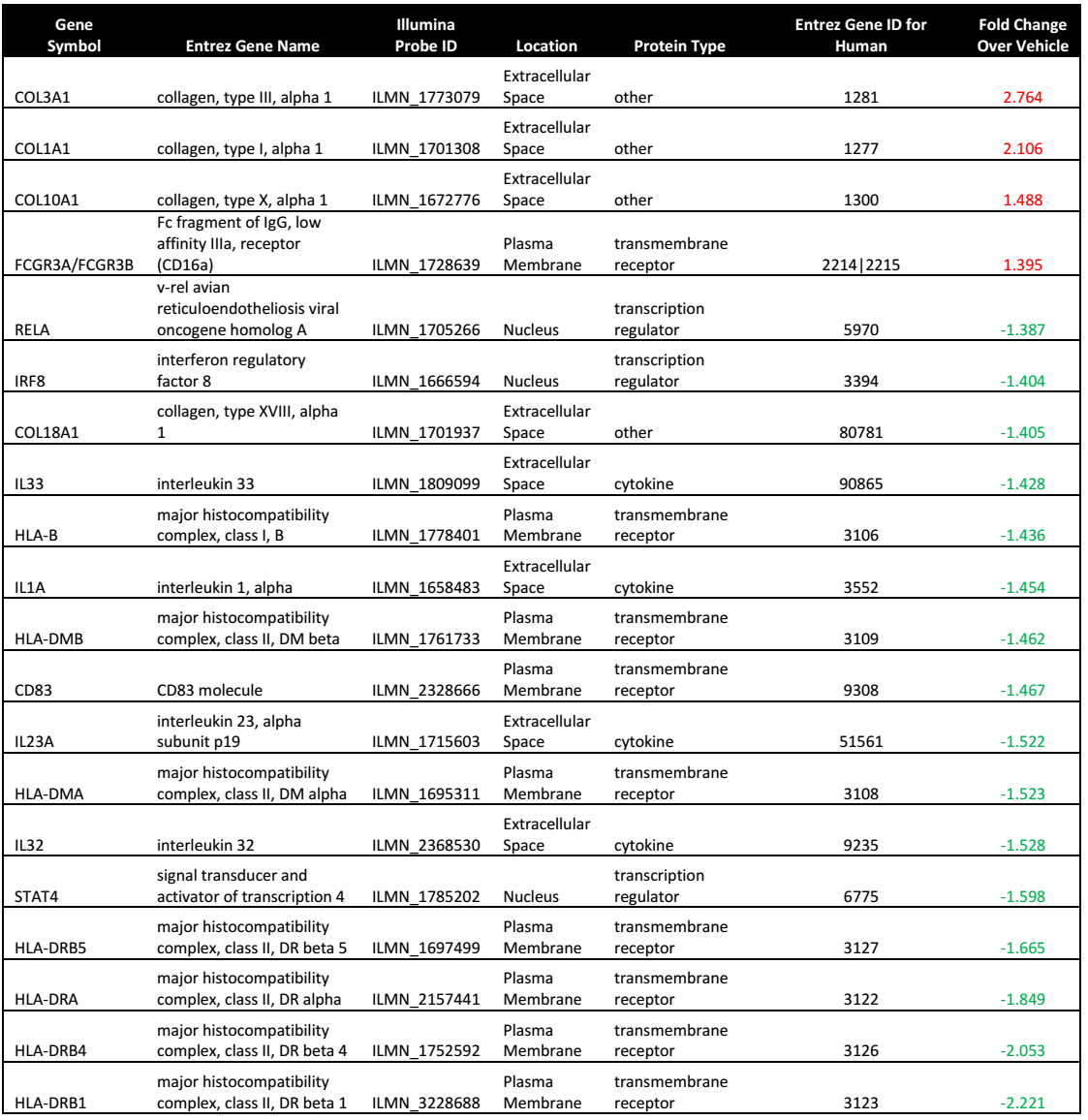

Supplement: Supplementary file 1 [file mmc1.docx]
